# Comparison of Functional and Radiological Outcomes of Intramedullary Nailing of the Tibia Using Suprapatellar and Infrapatellar Approaches

**DOI:** 10.7759/cureus.92645

**Published:** 2025-09-18

**Authors:** Anurag Agrawal, Rajeev Kelkar, Pranav Mahajan

**Affiliations:** 1 Orthopedics and Traumatology, Mahatma Gandhi Memorial (MGM) Medical College and Maharaja Yeshwantrao (MY) Hospital, Indore, IND

**Keywords:** functional outcome, infrapatellar approach, intramedullary nailing, radiological alignment, suprapatellar approach, tibial shaft fracture

## Abstract

Background: Tibial shaft fractures are among the most common long bone injuries, frequently resulting from road traffic accidents, falls, and sports injuries. Intramedullary nailing (IMN) has become the gold standard for managing these fractures, but debate persists regarding the optimal surgical approach: suprapatellar (SPN) or infrapatellar (IFN).

Methods: This prospective observational study was conducted at Mahatma Gandhi Memorial (MGM) Medical College and Maharaja Yeshwantrao (MY) Hospital, Indore, between October 2023 and September 2024. Sixty patients with closed or grade 1 compound tibial shaft fractures were enrolled and randomized equally into SPN and IFN groups. Surgical time, postoperative pain (visual analog scale scores), functional outcomes (Lysholm Knee Score), radiological alignment, and complications were assessed at two weeks, six weeks, and three months postoperatively.

Results: The study population showed male predominance (88.33%), with a mean age of 32.1 years (SPN) and 36.2 years (IFN). Road traffic accidents were the leading cause of injury. The SPN group had significantly longer surgical times (77.63 ± 8.50 minutes) compared to the IFN group (68.80 ± 4.43 minutes; p = 0.00000836), though a learning curve was noted. Postoperative pain and functional recovery were similar in both groups across all follow-ups. By three months, the majority of patients in both groups achieved excellent functional outcomes, and radiological alignment was comparable. Minimal complications were observed, with only one case of knee stiffness in the SPN group, successfully managed.

Conclusion: Both SPN and IFN approaches for tibial IMN offer comparable functional and radiological outcomes at three months. Although the SPN approach had a longer operative time initially, it did not significantly affect postoperative pain, fracture healing, or complication rates. Choice of approach may therefore be tailored based on surgeon preference, fracture location, and patient-specific considerations.

## Introduction

Tibial shaft fractures are among the most frequently encountered long bone injuries in orthopedic practice, with an increasing incidence attributed largely to road traffic accidents, sports injuries, and falls. Due to its subcutaneous position along the medial border and the relative paucity of soft tissue coverage, tibial fractures are prone to complications such as delayed union, nonunion, and infection, particularly following high-energy trauma [1]. Intramedullary nailing (IMN) has become the gold standard for treating these fractures, favored for its mechanical stability, minimally invasive nature, and facilitation of early mobilization. However, the optimal surgical approach for tibial nailing remains a topic of ongoing debate.

Traditionally, the infrapatellar (IFN) approach, requiring knee flexion, has been the standard technique for tibial nailing. While effective, this approach has been associated with certain drawbacks, including difficulty in fracture reduction, increased malalignment rates, and postoperative anterior knee pain [2].

More recently, the suprapatellar (SPN) approach, performed with the knee in a semi-extended position, has gained popularity. This technique offers potential advantages such as improved alignment, decreased operative time, reduced intraoperative fluoroscopy use, and possibly less postoperative anterior knee discomfort [3]. Nonetheless, concerns regarding iatrogenic injury to the patellofemoral joint and elevated intra-articular pressures persist.

Given the lack of definitive consensus on the superiority of either approach, this study aims to compare the functional and radiological outcomes of IMN performed using the SPN and IFN approaches. By systematically analyzing key parameters such as surgical duration, fracture alignment, postoperative pain, functional recovery, and complications, this research seeks to contribute valuable insights to guide surgical decision-making in the management of tibial shaft fractures.

## Materials and methods

Study design and inclusion & exclusion criteria

This prospective observational study was conducted at Mahatma Gandhi Memorial (MGM) Medical College and Maharaja Yeshwantrao (MY) Hospital, Indore, over a period of one year, from October 2023 to September 2024. The aim of the study was to compare the functional and radiological outcomes of intramedullary nailing of tibial shaft fractures performed using the suprapatellar and infrapatellar approaches. A total of 60 patients meeting the inclusion criteria were enrolled after obtaining informed consent. Patients were allocated using a quasi-randomization method based on hospital registration numbers (odd numbers to the suprapatellar group, and even numbers to the infrapatellar group). Clinical evaluation was done by the surgical team. No independent blinded assessor was used. The intramedullary nailing system used was the Nebula Tibial Intramedullary Nailing Set (Nebula Surgical Pvt. Ltd., Gujarat, India).

Patients included were those with closed or grade 1 compound fractures of the tibial shaft, presenting within three weeks of injury, and aged over 18 years. Patients with significant contralateral lower limb injuries, comorbidities preventing surgery, or pre-existing knee arthropathy were excluded from the study. All patients were initially stabilized following the Advanced Trauma Life Support (ATLS) protocols. Radiographic evaluation, including anteroposterior and lateral views of the knee, leg, and ankle, was performed. Patients fulfilling the inclusion criteria were admitted, counseled about the study, and included after obtaining written informed consent. Pre-anesthetic evaluation was completed prior to surgical intervention.

Surgical technique

**Figure 1:**
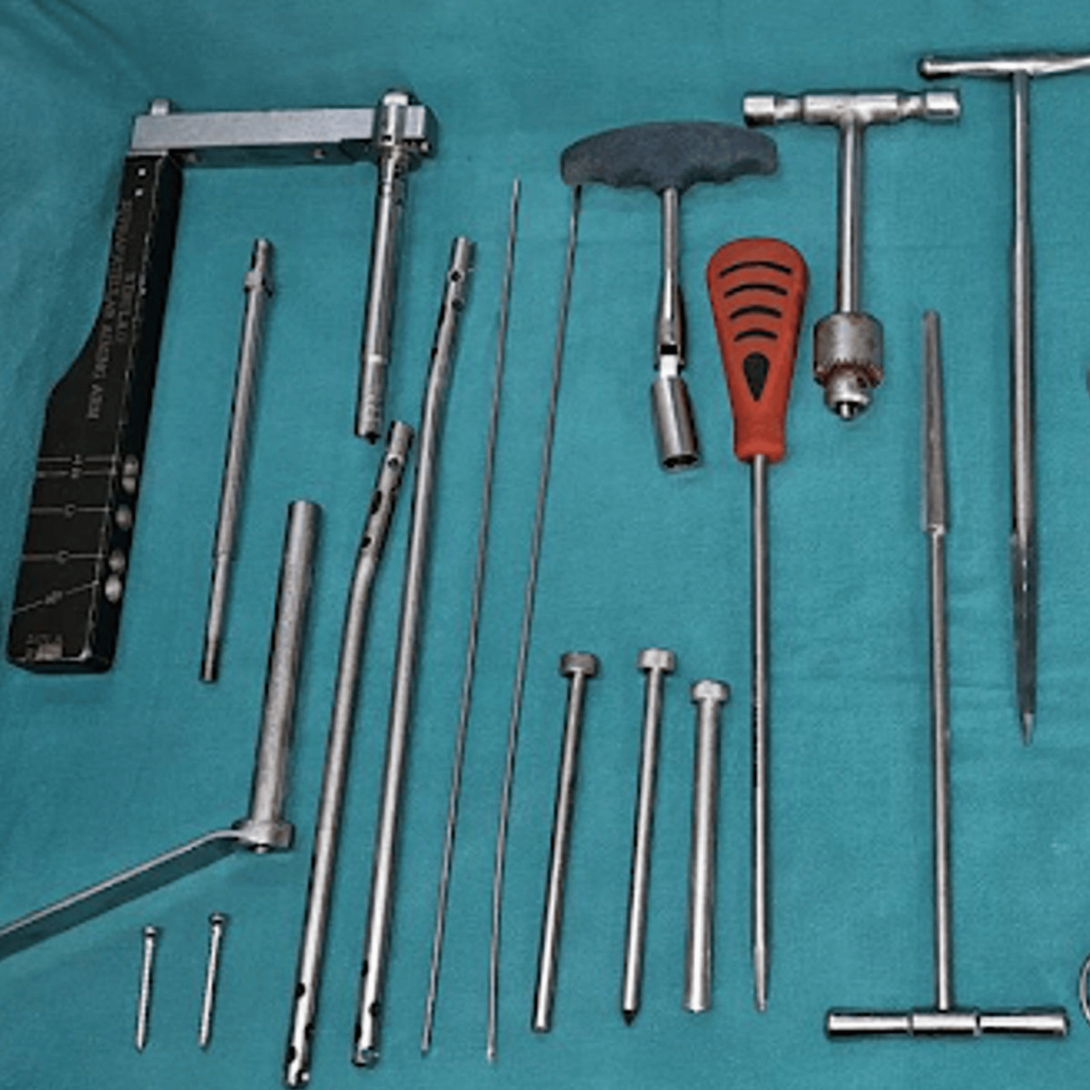
Instrumentation for suprapatellar nailing.

**Figure 2:**
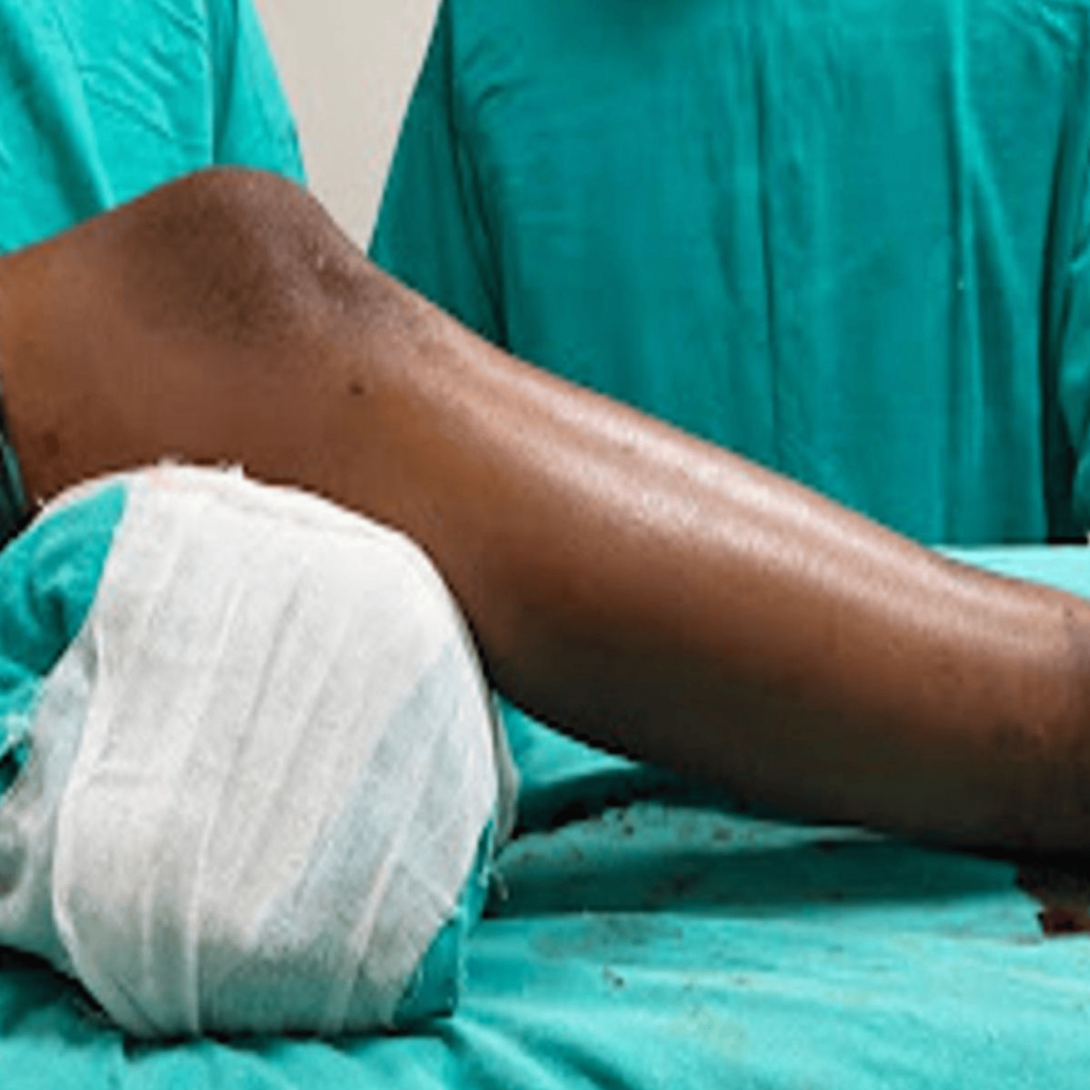
Semi-extended position of the knee in suprapatellar nailing.

**Figure 3:**
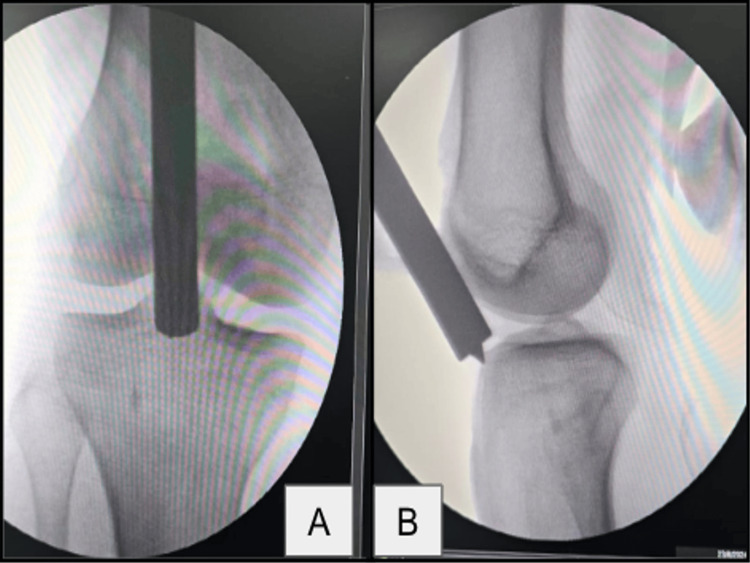
Entry point of the guide pin in the suprapatellar approach. (A) Anteroposterior view. (B) Lateral view.

Surgical procedures were performed under spinal anesthesia with the patient in a supine position on a radiolucent table, under fluoroscopic guidance. The instrumentation used is depicted in Figure 1. In the suprapatellar group, the knee was maintained in a semi-extended position, as shown in Figure 2, and a small midline incision was made proximal to the patella. Blunt dissection through the quadriceps tendon was performed, followed by hydrodissection with saline to release intra-articular adhesions. The entry point for the guidewire was established just lateral to the tibial tubercle in the anteroposterior view and just anterior to the articular margin in the lateral view, as shown in Figure 3. A protective metal sleeve was placed to shield the knee joint, and the guidewire was advanced under fluoroscopic control. Sequential reaming of the medullary canal was carried out, and the appropriate size of the intramedullary nail was inserted. Proximal locking was performed with a jig, while distal locking was achieved using a freehand technique under fluoroscopy. Thorough lavage of the joint was performed before closure.

In the infrapatellar group, the procedure was performed with the knee flexed to 90 degrees. A standard infrapatellar midline incision with tendon splitting was performed. The subsequent steps of guidewire insertion, reaming, nail insertion, and locking were identical to those used in the suprapatellar group.

Postoperative protocol and follow-up

Postoperatively, intravenous antibiotics were administered for three days, followed by oral antibiotics for five days (reflecting the standard practice at our institution and may vary between centers). Knee and ankle range of motion exercises were initiated from the first postoperative day to prevent stiffness and encourage early mobilization. Sutures were removed on the 14th postoperative day. Toe-touch and partial weight-bearing were allowed after suture removal, while full weight-bearing was initiated based on pain tolerance and radiographic signs of fracture union.

The intraoperative duration of surgery was recorded for all patients. Postoperative follow-up was conducted at two weeks, six weeks, and three months. Pain was assessed using the visual analog scale (VAS) in the immediate postoperative period, and at each follow-up visit (two weeks, six weeks, and three months). Functional outcomes were evaluated using the Lysholm Knee Scoring Scale [4] at two weeks, six weeks, and three months, with results categorized as excellent, good, fair, or poor. The interim Lysholm scores at two and six weeks were recorded mainly for supportive monitoring of recovery and early complications. Radiological evaluation was performed at three months using anteroposterior and lateral views to assess cortical alignment. Alignment was graded as excellent (four cortices aligned), good (three cortices), fair (two cortices), or poor (one cortex). For segmental fractures, alignment was graded as excellent (seven to eight cortices aligned), good (five to six cortices), fair (three to four cortices), or poor (one to two cortices). Postoperative complications, including knee stiffness, superficial or deep infection, screw loosening, need for re-operation, and joint infection, were systematically monitored during the follow-up period.

Statistical analysis was performed using SPSS version 26.0 (IBM Corp., Armonk, NY). Continuous variables, including operative time and VAS scores, were compared between groups using the independent Student’s t-test. Ordinal categorical variables, such as radiological alignment (graded as excellent, good, fair, or poor) and Lysholm knee score categories, were analyzed using the chi-square test. A p-value of <0.05 was considered statistically significant.

## Results

The demographic details of patients in both groups included in the study are listed in Table 1.

**Table 1:** Demographic details of patients in each group.

Variable	Suprapatellar group (n = 30)	Infrapatellar group (n = 30)	Total (n = 60)
Mean age (years)	32.1	36.2	34.1
Sex (male)	27 (90%)	26 (86.7%)	53 (88.3%)
Sex (female)	3 (10%)	4 (13.3%)	7 (11.7%)
Side of injury – left	16 (53.3%)	17 (56.7%)	33 (55%)
Side of injury – right	14 (46.7%)	13 (43.3%)	27 (45%)
Mechanism of injury – road traffic accident	24 (80%)	21 (70%)	45 (75%)
Mechanism of injury – assault	5 (16.7%)	7 (23.3%)	12 (20%)
Mechanism of injury – fall	1 (3.3%)	2 (6.7%)	3 (5%)
Fracture pattern – proximal third	14 (46.7%)	4 (13.3%)	18 (30%)
Fracture pattern – middle third	5 (16.7%)	17 (56.7%)	22 (36.7%)
Fracture pattern – distal third	8 (26.7%)	6 (20%)	14 (23.3%)
Fracture pattern – segmental	3 (10%)	3 (10%)	6 (10%)

**Figure 4:**
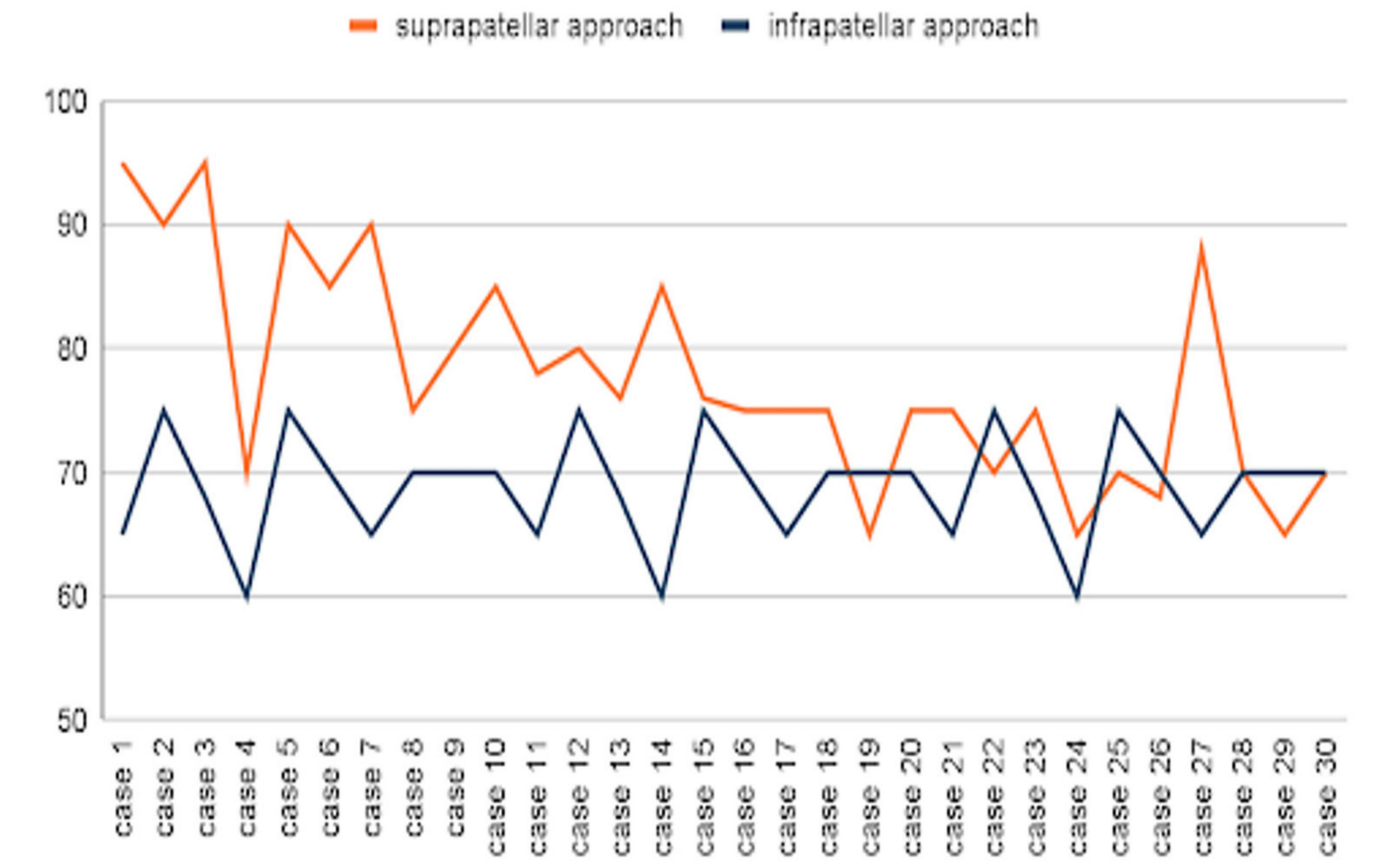
Duration of surgery in each approach.

The average duration of surgery in the suprapatellar group was 77.63 minutes (SD: 8.50 minutes), while in the infrapatellar group, it was 68.80 minutes (SD: 4.43 minutes). The mean surgical time between the two approaches was compared using an independent t-test, and it was statistically significant, i.e., p = 0.00000836 (p < 0.05 was considered significant). Additionally, a gradual decrease in surgery duration was observed in the suprapatellar group over time, indicating a learning curve effect at our center, as shown in Figure 4.

Postoperative pain was assessed using the VAS at multiple intervals. The mean VAS scores in both groups showed a gradual decline over time, indicating consistent pain reduction. Statistical analysis was done using an independent t-test, and a p-value of <0.05 was considered statistically significant. Immediately after surgery, the suprapatellar group had a slightly higher mean VAS score (5.7 ± 0.70) compared to the infrapatellar group (5.4 ± 0.62), although this difference was not statistically significant (p = 0.085). At two weeks, pain levels remained comparable between the groups, with mean VAS scores of 3.2 ± 0.55 in the suprapatellar group and 3.3 ± 0.95 in the infrapatellar group (p = 0.620). At six weeks, further pain relief was evident, with VAS scores of 1.93 ± 0.58 and 1.90 ± 0.76, respectively (p = 0.849). By three months, the mean VAS scores were close to zero in both groups (0.43 ± 0.50 for the suprapatellar group and 0.53 ± 0.51 for the infrapatellar group), confirming that postoperative pain had largely subsided (p = 0.447). The data are shown in Table 2.

**Table 2:** Statistical analysis of visual analog scale in each group. SD: standard deviation. An independent Student’s t-test was used. Significance: p < 0.05.

Time interval	Suprapatellar (n = 30), mean ± SD	Infrapatellar (n = 30), mean ± SD	P-value (independent Student’s t-test)
Immediate postoperative	5.7 ± 0.70	5.4 ± 0.62	0.085 (not significant)
At 2 weeks	3.2 ± 0.55	3.3 ± 0.95	0.620 (not significant)
At 6 weeks	1.93 ± 0.58	1.90 ± 0.76	0.849 (not significant)
At 3 months	0.43 ± 0.50	0.53 ± 0.51	0.447 (not significant)

Functional outcomes were primarily assessed using the Lysholm Knee Scoring Scale at three months postoperatively. Interim follow-ups at two weeks and six weeks were conducted to evaluate progress and detect any early complications, with functional scores noted for descriptive comparison. At two weeks, most cases in both groups fell within the fair category, although the infrapatellar group had a slightly higher proportion of poor outcomes; the difference was not statistically significant (p = 0.561). By six weeks, there was a marked shift toward the good category in both groups, again without a significant difference (p = 0.356). At the three-month assessment, the majority of patients in both groups achieved excellent scores, with no cases remaining in the poor category (p = 0.734), suggesting similar functional recoveries between the two approaches. Data for the same are shown in Table 3.

**Table 3:** Statistical analysis of Lysholm score in each group. Values are expressed as n (%). Chi-square test was used for statistical analysis. P-value <0.05 is considered significant. SPN: suprapatellar; IFN: infrapatellar.

Time point	Category	SPN (n = 30)	IFN (n = 30)	P-value (chi-square test)
2 weeks	Excellent	0 (0%)	0 (0%)	0.561 (not significant)
	Good	2 (6.7%)	2 (6.7%)	
	Fair	21 (70.0%)	15 (50.0%)	
	Poor	7 (23.3%)	13 (43.3%)	
6 weeks	Excellent	1 (3.3%)	1 (3.3%)	0.356 (not significant)
	Good	15 (50.0%)	17 (56.7%)	
	Fair	13 (43.3%)	11 (36.7%)	
	Poor	1 (3.3%)	1 (3.3%)	
3 months	Excellent	21 (70.0%)	19 (63.3%)	0.734 (not significant)
	Good	8 (26.7%)	10 (33.3%)	
	Fair	1 (3.3%)	1 (3.3%)	
	Poor	0 (0%)	0 (0%	

Radiological evaluation was performed at three months postoperatively using the alignment of cortices on anteroposterior and lateral views, assessing the continuity of each cortex. Alignment was graded as excellent (four cortices aligned), good (three cortices aligned), fair (two cortices aligned), and poor (one cortex aligned). For segmental fractures, scores were graded as excellent (seven to eight cortex aligned), good (five to six cortex aligned), fair (three to four cortex aligned), and poor (one to two cortex aligned). In the suprapatellar group, 18 cases (60.0%) demonstrated excellent alignment, nine cases (30.0%) showed good alignment, and three cases (10.0%) showed fair alignment; no cases were graded as poor. In the infrapatellar group, 15 cases (50.0%) demonstrated excellent alignment, 10 cases (33.3%) showed good alignment, four cases (13.3%) showed fair alignment, and one case (3.3%) was graded as poor. Although the suprapatellar group demonstrated a slightly higher proportion of excellent alignments, the difference between the groups was not statistically significant (χ²(3) = 1.29, p = 0.732). Data for the same are shown in Table 4.

**Table 4:** Radiological alignment at three months in each group. Values are presented as n (%). Chi-square test was used for statistical analysis. P-value < 0.05 is considered significant.

Alignment grade at 3 months	Suprapatellar (n = 30)	Infrapatellar (n = 30)	P-value, chi-square test
Excellent (4 cortices)	18 (60.0%)	15 (50.0%)	0.732 (not significant)
Good (3 cortices)	9 (30.0%)	10 (33.3%)	
Fair (2 cortices)	3 (10.0%)	4 (13.3%)	
Poor (1 cortex)	0 (0%)	1 (3.3%)	

Postoperative complications were monitored during follow-up visits at two weeks, six weeks, and three months. Common complications assessed included knee stiffness, infection, screw loosening, joint infection, and the need for re-operation. The incidence of complications was minimal. There were no cases of superficial or deep infection, screw loosening, joint infection, or re-operation in either group.

**Figure 5:**
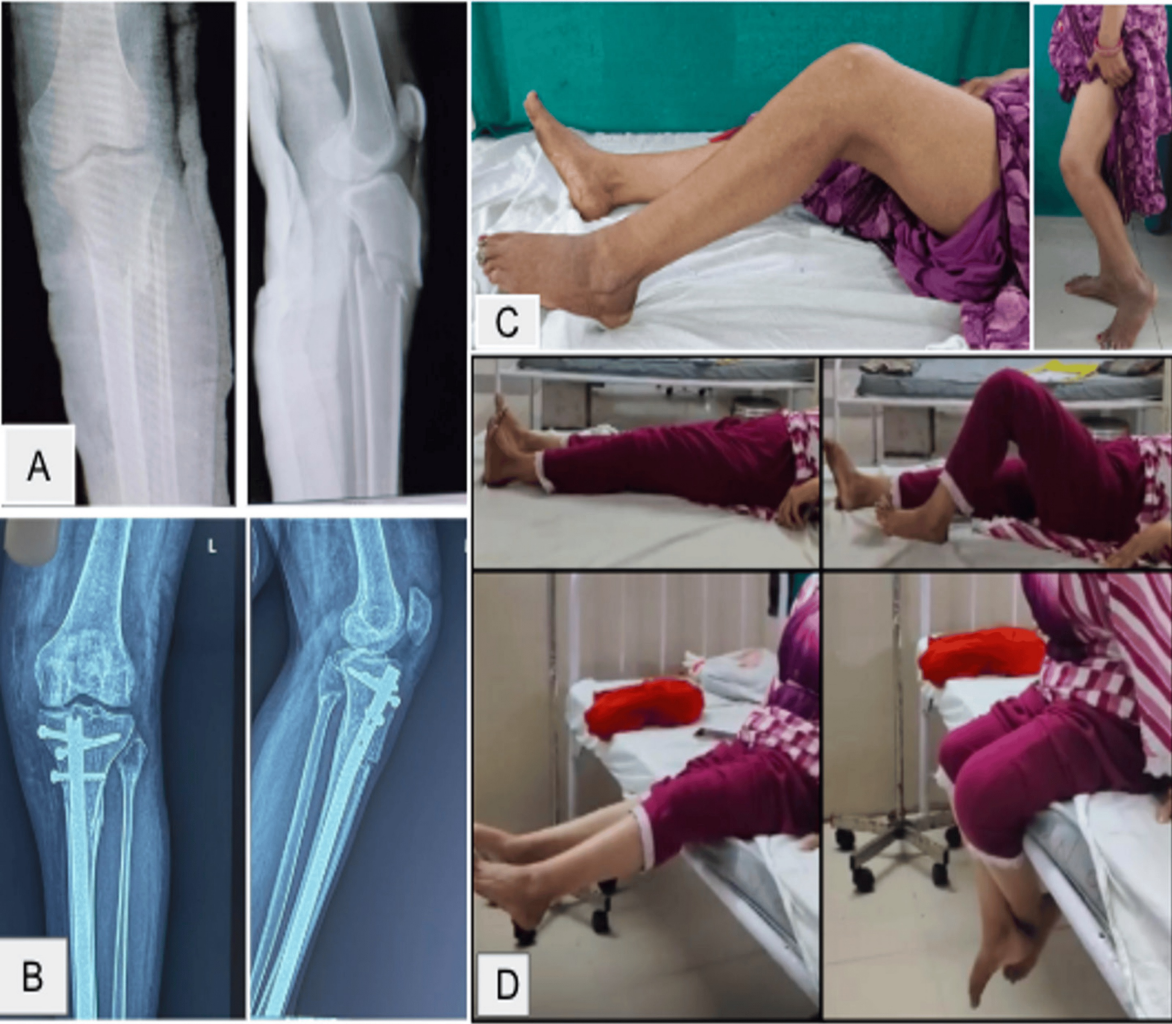
Case of knee stiffness in the suprapatellar group. (A) Preoperative X-ray. (B) X-rays at three months postoperative. (C) Restriction of knee flexion at three months (before adhesiolysis). (D) Knee range of motion attained after adhesiolysis.

Only one patient in the suprapatellar group developed knee stiffness at the three-month follow-up, with knee flexion restricted to 20°. This was successfully managed with adhesiolysis of the anterior and anteromedial fibers of the quadriceps tendon, following which the patient achieved 110° of knee flexion, as shown in Figure 5.

**Figure 6:**
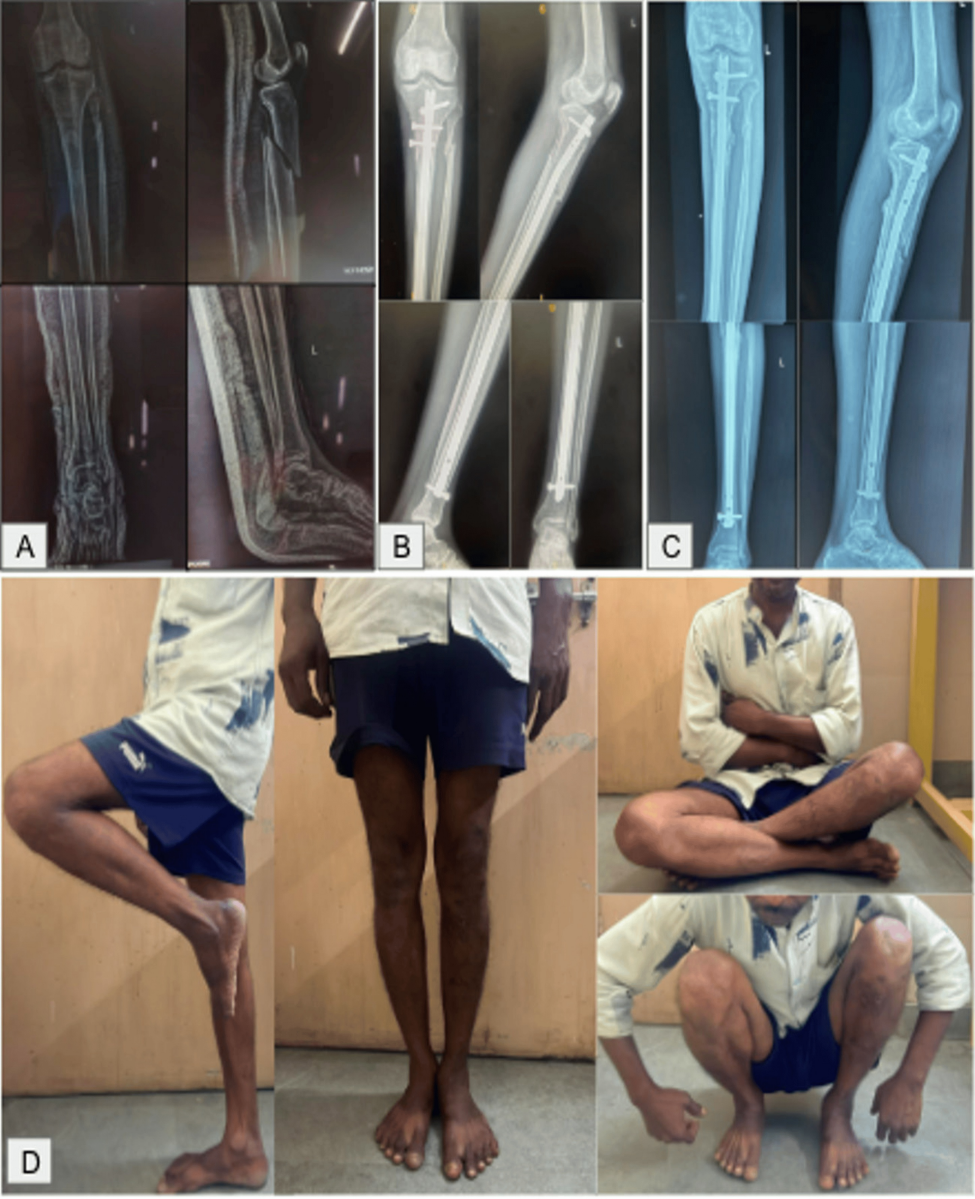
Case 1 of suprapatellar nailing. (A) Preoperative X-ray. (B) Immediate postoperative X-rays. (C) Three months postoperative X-rays. (D) Clinical assessment and knee range of motion at three months.

**Figure 7:**
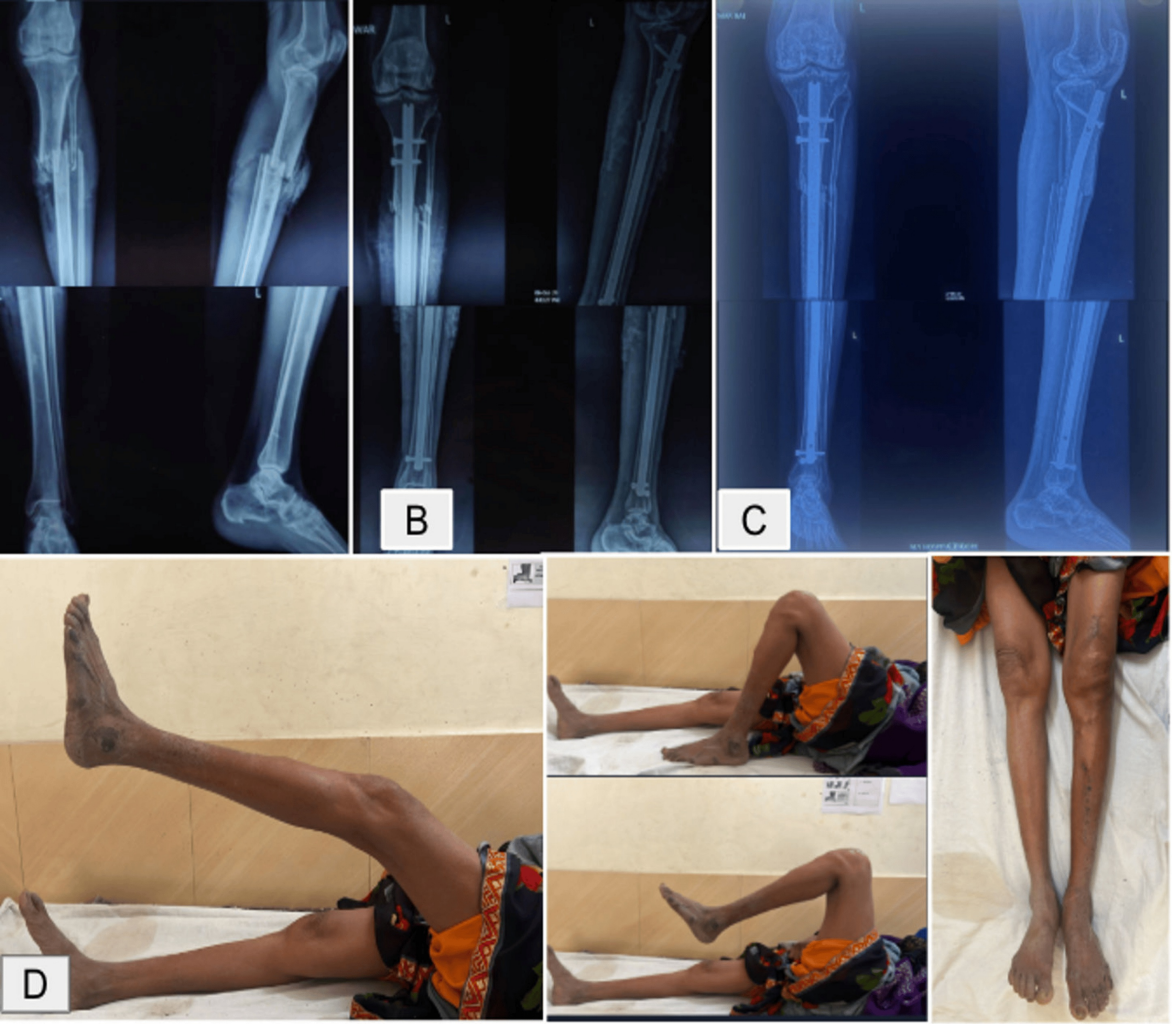
Case 2 of suprapatellar nailing. (A) Preoperative X-ray. (B) Immediate postoperative X-rays. (C) Three months postoperative X-rays. (D) Clinical assessment and knee range of motion at three months.

**Figure 8:**
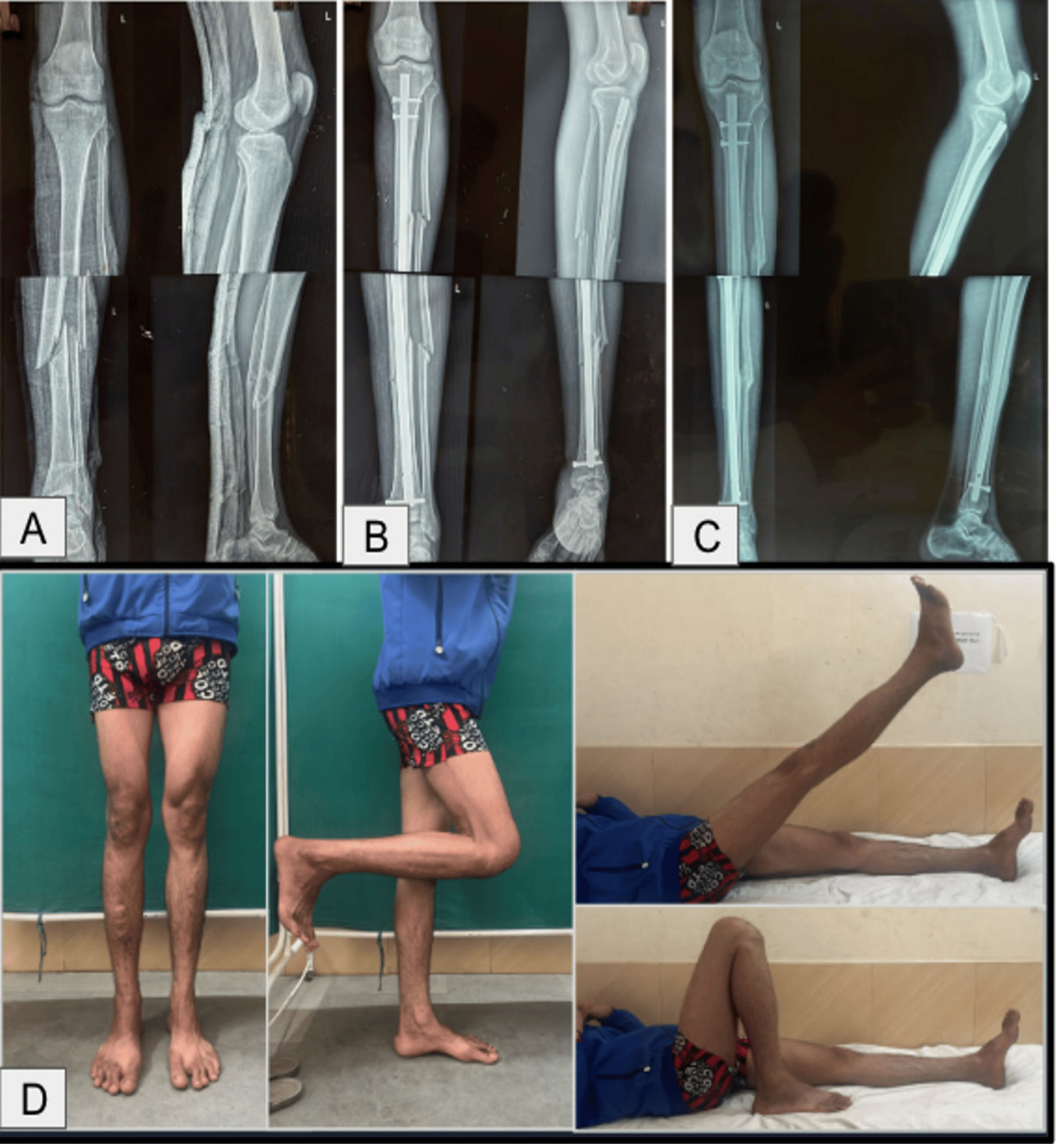
Case 1 of infrapatellar nailing. (A) Preoperative X-ray. (B) Immediate postoperative X-rays. (C) Three months postoperative X-rays. (D) Clinical assessment and knee range of motion at three months.

**Figure 9:**
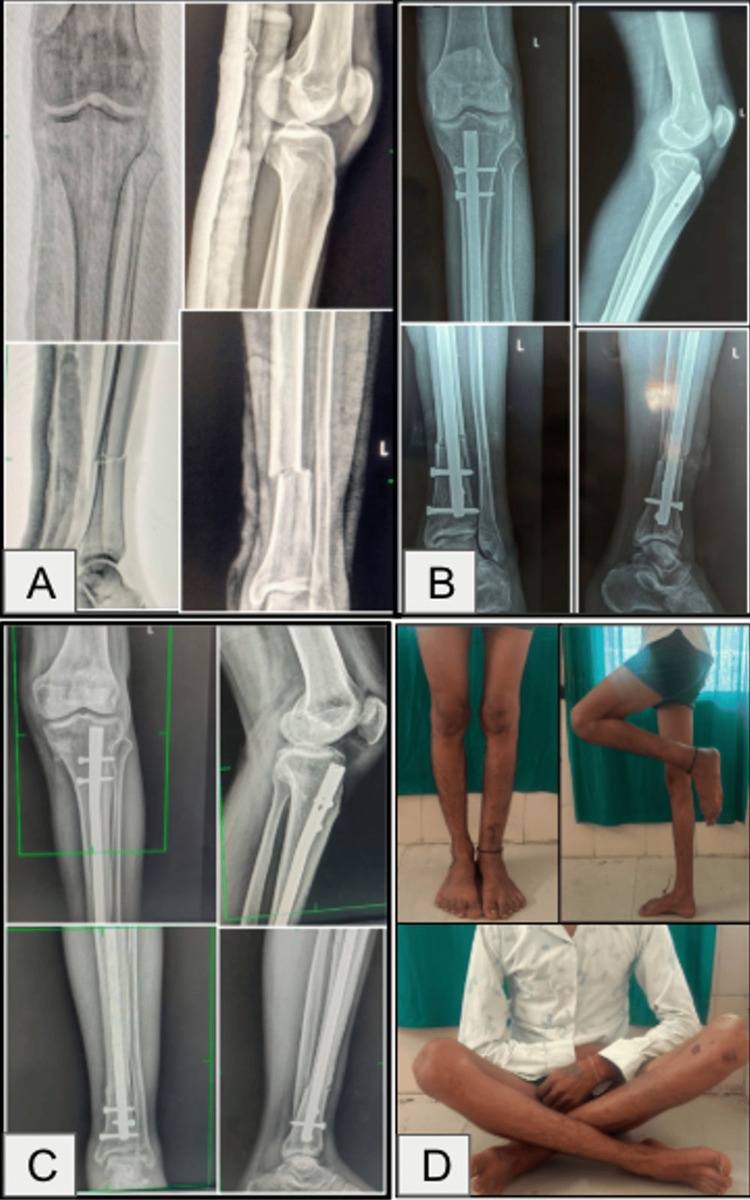
Case 2 of infrapatellar nailing. (A) Preoperative X-ray. (B) Immediate postoperative X-rays. (C) Three months postoperative X-rays. (D) Clinical assessment and knee range of motion at three months.

Representative clinical photographs of four cases are shown in Figures 6-9. These include preoperative, immediate postoperative, and follow-up X-rays at three months, and clinical photos showing functional recovery at final follow-up. All radiographs shown correspond to the three-month follow-up. Some patients, particularly younger adults with good bone biology, demonstrated early cortical consolidation and partial remodeling within this period.

## Discussion

Our study demonstrated a significant male predominance, with 88.33% of the patients being male and 11.67% female, resulting in a male-to-female ratio of 7.57:1. This finding aligns with previous literature [5], indicating a higher incidence of tibial fractures among males, attributed to greater exposure to road traffic accidents (RTAs), assault, and trauma due to falls.

The mean age of patients was slightly lower in the SPN group (32.1 years) compared to the IFN group (36.2 years), consistent with the literature, suggesting that younger adults are more prone to high-energy injuries. The majority of fractures were left-sided (n = 33, 55%), with a similar distribution across both groups. Although the cause of laterality remains unclear, it may relate to dominant limb reflexes during trauma. However, laterality did not significantly influence surgical outcomes or healing patterns. RTAs accounted for 75% (n = 45) of the injuries, with a slightly higher proportion observed in the SPN group (n = 24, 80%) compared to the IFN group (n = 21, 70%). Assault (n = 12, 20%) and falls (n = 3, 5%) were less common mechanisms, reflecting the high-energy nature of most tibial fractures in our study population.

Fracture pattern distribution revealed a higher proportion of proximal third fractures in the SPN group (n = 14, 46.67%), consistent with the preference for the SPN approach in managing proximal tibial fractures [3], where maintaining alignment can be challenging. In contrast, the IFN group had a higher proportion of middle third fractures (n = 17, 56.67%), which are typically more manageable using the traditional infrapatellar technique. Distal third and segmental fractures were comparable between the groups.

Operative time was significantly longer in the SPN group (77.63 ± 8.50 minutes) compared to the IFN group (68.80 ± 4.43 minutes), with a significant p-value of 0.00000836. This finding supports the observations made by Wang et al. [6] and Xu et al. [7], who noted longer surgical durations for the SPN approach, particularly during the initial learning phase. However, our study also observed a learning curve effect, with operative times gradually decreasing as surgical experience increased, echoing the findings of Al-Azzawi et al. [8].

Pain assessment using the VAS demonstrated comparable postoperative pain levels between the groups. Immediate postoperative pain was slightly higher in the SPN group (5.7 ± 0.70) compared to the IFN group (5.4 ± 0.62), but the difference was not statistically significant (p = 0.085). Pain scores continued to decline over time and remained similar at two weeks, six weeks, and three months, consistent with Ponugoti et al. [9]. Although Yang et al. [10] suggested that the SPN approach may result in reduced long-term anterior knee pain, this trend was not evident within the short-term follow-up of our study.

Functional outcomes were assessed using the Lysholm Knee Score at three months, which served as the primary endpoint. Interim follow-ups at two weeks and six weeks were conducted mainly to evaluate clinical progress and identify any early complications, with functional scores recorded for descriptive comparison. At two weeks, most cases in both groups fell within the fair category, although the infrapatellar group had a slightly higher proportion of poor outcomes (13 patients (43.3%) vs. seven patients (23.3%) in SPN; p = 0.561). By six weeks, there was a marked shift toward the good category in both groups, with 16 patients (53.3%) in the SPN group and 18 patients (60.0%) in the IFN group achieving good to excellent results, and only one patient (3.3%) in each group remaining in the poor category (p = 0.356). At the three-month assessment, excellent outcomes predominated (21 patients (70.0%) in SPN vs. 19 patients (63.3%) in IFN), with most of the remainder classified as good (eight patients (26.7%) in SPN vs. 10 patients (33.3%) in IFN), and no cases in the poor category (p = 0.734). These findings suggest comparable functional recovery between the two approaches. Results are consistent with Xu et al. [7] and Al-Azzawi et al. [8]. While Wang et al. [6] suggested that anterior knee pain associated with the IFN approach may influence long-term function, such effects were not evident within the follow-up period of our study.

Radiological outcomes assessed by cortical alignment at three months showed that in the SPN group, 18 patients (60.0%) demonstrated excellent alignment, nine patients (30.0%) had good alignment, and three patients (10.0%) showed fair alignment, with none in the poor category. In the IFN group, 15 patients (50.0%) demonstrated excellent alignment, 10 patients (33.3%) had good alignment, four patients (13.3%) showed fair alignment, and one patient (3.3%) demonstrated poor alignment. Although the SPN group had a slightly higher proportion of excellent alignment and no cases of poor alignment compared to the IFN group, the difference between groups was not statistically significant (p = 0.732). These results suggest that both suprapatellar and infrapatellar approaches provide comparable radiological alignment outcomes. Similar findings have been reported by Ponugoti et al. [9] and Yang et al. [10], who also demonstrated no significant difference in radiological healing between the two techniques.

Table 5 summarizes the results. Both suprapatellar and infrapatellar approaches yielded comparable short-term functional and radiological outcomes. Although the SPN approach initially required longer operative time and showed a learning curve, it demonstrated favorable results, particularly for proximal tibial fractures, without an increase in postoperative complications.

**Table 5:** Summary of outcomes in each group. Values are mean ± standard deviation or n (%). Statistical analysis was done through the test mentioned. P < 0.05 is considered significant. VAS: visual analog scale.

Parameter	Suprapatellar (SPN, n = 30)	Infrapatellar (IFN, n = 30)	Statistical test used	P-value	Significance
Operative time (minutes)	77.6 ± 8.5	68.8 ± 4.4	Independent t-test	0.000008	Significant
VAS (at 3 months)	Gradual ↓ to 0.43	Gradual ↓ to 0.53	Independent t-test	0.447	Not significant
Lysholm score (at 3 months)	Majority excellent/good	Majority excellent/good	Chi-square test	0.734	Not significant
Radiological alignment (at 3 months)	Majority excellent/good	Majority excellent/good	Chi-square test	0.732	Not significant

Postoperative complications were minimal in our study. No cases of superficial or deep infection, screw loosening, joint infection, or re-operation were reported in either group. Only one case of knee stiffness was observed in the SPN group. This contrasts with Wang et al. [6], who reported a higher incidence of anterior knee pain and stiffness with the IFN approach. Our findings are more in line with Xu et al. [7] and Ponugoti et al. [9], who observed no significant difference in complication rates between the two approaches. Additionally, Yang et al. [10] suggested that the SPN approach may lower infection risk due to reduced soft tissue disruption, a potential advantage that could not be conclusively evaluated in our study due to the low overall complication rates.

The limitations of our study include the relatively small sample size of 60 patients and being conducted at a single tertiary care center, which may restrict generalizability. The study used quasi-randomization due to institutional limitations. The follow-up period was limited to three months, preventing assessment of long-term complications such as chronic anterior knee pain, patellofemoral cartilage degeneration, and late functional decline. Operative time in the suprapatellar group was influenced by the surgical learning curve, which may have biased the comparison. Fluoroscopy time was not evaluated systematically. Additionally, as this was not a blinded study, observer bias in functional outcome assessment cannot be excluded.

## Conclusions

Our study compared the SPN and IFN approaches for tibial intramedullary nailing and found comparable short-term outcomes in terms of postoperative pain, functional recovery, radiological alignment, and complication rates. Although the SPN approach demonstrated a significantly longer operative time (p = 0.000008; statistically significant), this difference decreased with increasing surgical experience, suggesting a learning curve effect. Postoperative pain, assessed using the VAS, showed a gradual decline in both groups with no statistically significant difference (p = 0.447; not statistically significant) at any follow-up. Functional recovery, measured using the Lysholm Knee Scoring Scale at three months (p = 0.734; not statistically significant), was comparable between the groups. Radiological alignment also showed no statistically significant difference between the two techniques (p = 0.732; not statistically significant). The overall complication rate was low, with only one case of knee stiffness in the SPN group and no cases of infection, screw loosening, joint infection, or re-operation in either group.

When compared with existing literature, our findings align with previous meta-analyses and clinical studies, reinforcing that both approaches are viable options for tibial nailing. The choice of technique may ultimately depend on the surgeon's preference, familiarity, and experience. Despite the limitations, our study provides prospective data from a tertiary care center in central India, specifically addressing the early postoperative period, a timeframe that is often underreported in the literature. This information is valuable for surgeons practicing in similar settings, where early recovery, the surgical learning curve, and short-term complications can significantly influence clinical decision-making. By highlighting these early outcomes, our study complements existing long-term data and supports informed surgical choices for tibial nailing.
